# Comparative Analysis of Metabolomic Responses in On-Pump and Off-Pump Coronary Artery Bypass Grafting

**DOI:** 10.5761/atcs.oa.24-00126

**Published:** 2024-12-05

**Authors:** Chananya Karunasumetta, Wijittra Tourthong, Rachata Mala, Chotika Chatgasem, Theerayut Bubpamala, Suriya Punchai, Kittisak Sawanyawisuth

**Affiliations:** 1 Division of Cardiovascular and Thoracic Surgery, Department of Surgery, Faculty of Medicine, Khon Kaen University, Khon Kaen, Thailand; 2 Khon Kaen University National Phenome Institute, Khon Kaen University, Khon Kaen, Thailand; 3 Department of Surgery, Faculty of Medicine, Khon Kaen University, Khon Kaen, Thailand; 4 Department of Medicine, Faculty of Medicine, Khon Kaen University, Khon Kaen, Thailand

**Keywords:** on-pump CABG, off-pump CABG, metabolomic study, differential metabolites

## Abstract

**Purpose:** Although the clinical outcomes of on-pump (ONCAB) and off-pump CABG (OPCAB) are well established, their metabolomic impacts remain underexplored. This study aims to compare the metabolic profiles of ONCAB and OPCAB to identify differential metabolites associated with clinical outcomes.

**Methods:** In a prospective cohort study conducted between January 2023 and September 2023, 100 plasma samples from 20 patients undergoing isolated elective CABG (10 per group) were analyzed. Samples were collected preoperatively and at multiple postoperative time points (Days 0–3) and processed using proton nuclear magnetic resonance (^1^H-NMR). Advanced statistical modeling was applied to identify differential metabolites.

**Results:** No significant differences were found in clinical outcomes, although ONCAB showed higher postoperative CKMB levels. Both procedures induced metabolomic alterations, with ONCAB demonstrating a more substantial impact, particularly on Day 0. Key metabolites, including leucine, succinate, creatine, glucose, and adenine, affected starch and sucrose metabolism.

**Conclusion:** ONCAB induces more pronounced metabolic shifts immediately postsurgery, involving protein and energy turnover, oxidative stress, and disrupted glucose metabolism, indicative of cellular stress responses. A comprehensive understanding of these metabolic changes is critical for informing targeted interventions and supports the use of OPCAB as a preferred strategy for patients with elevated metabolic risks.

## Introduction

Coronary artery bypass grafting (CABG) is a common cardiac procedure performed using On-Pump (ONCAB) or Off-Pump (OPCAB) techniques. Extensive research compares their clinical outcomes, with OPCAB showing promise in reducing complications related to cardiopulmonary bypass (CPB) and aortic cross-clamping, such as low cardiac output syndrome, acute kidney injury, and postoperative stroke. Several landmark studies, including the CORONARY trial,^1)^ have shown that OPCAB and ONCAB yield comparable long-term survival and outcomes, supporting the idea that both approaches can be effective when carefully tailored to clinical circumstances. However, other studies, such as the ROOBY trial,^2)^ report contradictory findings, associating OPCAB with higher re-intervention rates and lower survival in certain cases. These differences emphasize the importance of patient-specific factors and surgeon experience in determining the optimal surgical approach.

While clinical outcomes are well-studied, the impact of these techniques on metabolomic responses is less understood. Therefore, we employed metabolomics to investigate dynamic shifts in metabolomic profiles during ONCAB and OPCAB, providing real-time insights into cellular activity.^3)^ Understanding these metabolomic pathways can elucidate surgery-related pathophysiology, which is related to clinical courses and complications. Our study aims to compare the metabolic profiles of ONCAB and OPCAB, identifying specific differential metabolites that may correlate with patient outcomes and prognostic indicators.

## Materials and Methods

### Study design and population

The protocol for this study was approved by the Ethical Committee of the Center for Ethics in Human Research at the Faculty of Medicine, Khon Kaen University. Between January 2023 and September 2023, we conducted a prospective cohort study at Srinagarind Hospital and Queen Sirikit Heart Center of the Northeast. Twenty eligible patients were recruited, with ten in the ONCAB group and another ten in the OPCAB group. The sample size was based on a prior study^4)^ to detect metabolite differences between groups. The selection of CABG techniques depended on the surgeon’s preference and the patient’s clinical condition. Inclusion criteria were patients over 18 scheduled for isolated CABG. Exclusion criteria included poorly controlled diabetes (HbA1c >8 or insulin use), serum creatinine >2 mg/dL, recent myocardial infarction, immune diseases, cancer, or the need for emergency surgery.

### Operative procedure and sample collection

**Fig. 1:**
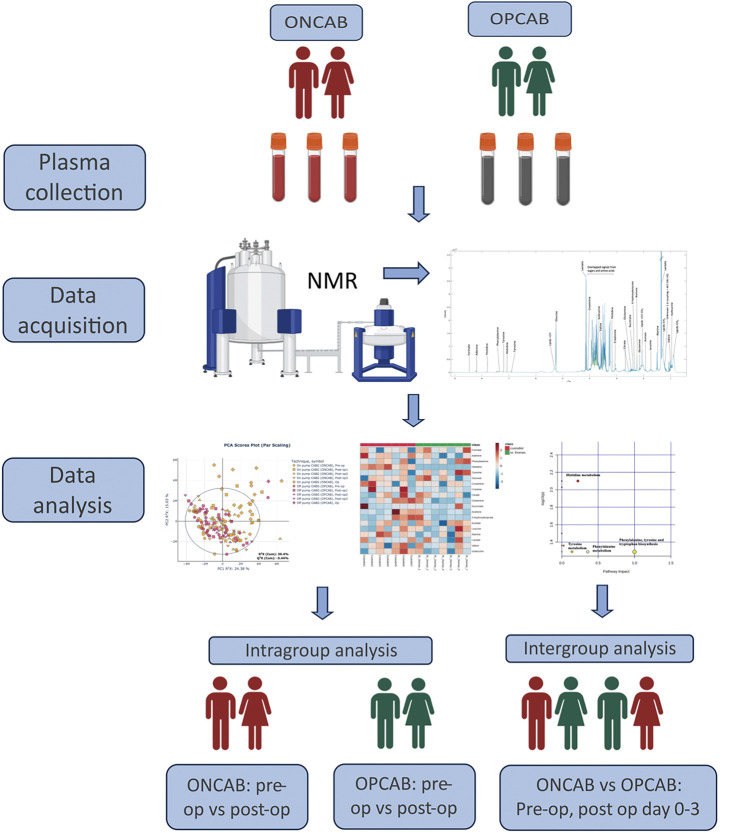
The study flow. CABG: coronary artery bypass graft; ONCAB: on-pump CABG; OPCAB: off-pump CABG; NMR: nuclear magnetic resonance spectroscopy. Created with BioRender.com

The techniques were uniform and performed by experienced CABG surgeons. Both ONCAB and OPCAB procedures involved a median sternotomy. For ONCAB, CPB was established via the ascending aorta and a right atrial cannula. Cardiac arrest was induced with aortic cross-clamping and antegrade blood-based cardioplegia. All patients received a left internal mammary artery graft for anastomosis to the left anterior descending artery. Additional arterial or venous grafts were used for other target vessels. After distal anastomosis, the aortic cross-clamp was removed, and proximal anastomosis was completed with partial aortic clamping during a beating heart. OPCAB replicated ONCAB but without CPB. Blood samples were collected preoperatively and on postoperative Days 0–3. Two milliliters of blood samples were collected into heparinized tubes (VACUETTE; Greiner Bio-One, Kremsmünster, Austria), centrifuged at 9000 rpm for 15 min at 4°C, and plasma aliquots were stored at −80°C for future analysis (**Fig. 1**).

### Data acquisition for proton nuclear magnetic resonance (^1^H-NMR) analysis

Samples for ^1^H-NMR analysis were prepared by mixing 400 µL of plasma with 400 µL of a phosphate buffer in D_2_O (pH 7.4) containing sodium azide and trimethylsilylpropanoic acid (TSP) as a reference. After vortexing and ultrasonication, samples were centrifuged, and 600 µL of the clear supernatant was transferred to NMR tubes. High-resolution spectra were acquired using a 400 MHz NMR spectrometer with a CPMG pulse sequence. Spectral processing included phase and baseline corrections, excluding the water signal. STOCSY facilitated metabolite identification and validated it against databases and literature. MATLAB was used for peak integration, with TSP ensuring internal calibration of peak concentrations.

### Statistical analysis

Continuous data normality was assessed using the Shapiro–Wilk test. Normally distributed data were analyzed using mean and standard deviation (SD) with one-way analysis of variance, while non-normal data were analyzed using median and interquartile range with the Kruskal–Wallis test. Categorical data were presented as numbers and percentages and analyzed using Pearson’s chi-squared test or Fisher’s exact test. Generalized estimating equations (GEE) were employed to evaluate biological markers involving repeated measurements and inotropic drug use.

### Univariate and multivariate statistical analysis of NMR spectral data

Data analysis, modeling, evaluation, and visualization were conducted using SIMCA software 14, Python (version 3.12.1, https://www.python.org), and associated packages: pandas (version 1.1.3, https://pandas.pydata.org), NumPy (version 1.19.2, https://numpy.org), and the GitHub repository (https://github.com/aeiwz/metbit). Visual studio code (https://code.visualstudio.com) was utilized for development. Model validity was assessed through rigorous cross-validation and permutation testing (P <0.05). Orthogonal partial least squares discriminant analysis (OPLS-DA) identified relevant variables with P (corr) ≥|0.5| and VIP score >2.0 for discriminating between the groups. Absolute concentrations of candidate metabolites were determined relative to known concentrations of TSP using a formula detailed in Phetcharaburanin et al. (2020).^5)^ Differential metabolites were further analyzed using the Mann–Whitney *U* test in (SPSS version 25; IBM, Armonk, NY, USA) with significance set at P <0.05. Metabolic profiles were visualized using heatmap analysis, and metabolic pathway analysis was performed using MetaboAnalyst 6.0 (http://www.metabo-analyst.ca). Spearman’s correlation coefficient assessed the relationships between metabolite abundances and cardiac enzymes.^6)^

## Results

The demographic data of patients are presented in **Table 1**. Overall, there were no significant differences between the groups. Additionally, there were no significant differences in preoperative lab chemistry.

**Table 1:** Demographic data of the patient

	Total (n = 20)	Technique		P-value
		OPCAB (n = 10)	ONCAB (n = 10)	
Age (years); median (IQR)	66 (60, 73)	67 (57, 73)	66 (65, 72)	0.894
Sex, male (%)	13 (65.00)	7 (70.00)	6 (60.00)	>0.999
BMI; mean (SD)	25.08 (3.35)	23.75 (3.44)	26.41 (2.82)	0.075
DM (%)	15 (75.00)	7 (70.00)	8 (80.00)	>0.999
HT (%)	17 (85.00)	8 (80.00)	9 (90.00)	>0.999
Dyslipidemia (%)	17 (85.00)	8 (80.00)	9 (90.00)	>0.999
Stroke (%)	1 (5.00)	1 (10.00)	–	>0.999
Smoking (%)	4 (20.00)	4 (40.00)	–	0.087
Alcohol (%)	4 (20.00)	3 (30.00)	1 (10.00)	0.582
EURO score (%); median (IQR)	0.96 (0.73, 1.2)	0.86 (0.69, 1.16)	1.03 (0.82, 1.24)	0.739
LVEF by echo; mean (SD)	53.4 (14.93)	51.7 (16.96)	55.09 (13.29)	0.625
Hemoglobin (g/dL); mean (SD)	11.98 (1.55)	11.83 (1.67)	12.12 (1.49)	0.687
Blood sugar (mg/dL); mean (SD)	159.8 (61.72)	156.1 (43.24)	163.5 (78.37)	0.797
HbA1C (%); mean (SD)	7.38 (1.25)	7.5 (1.38)	7.25 (1.16)	0.666
Creatinine (mg/dL); median (IQR)	1.12 (0.77, 1.41)	1.11 (0.97, 1.35)	1.18 (0.7, 1.46)	0.986
Albumin (g/dL); mean (SD)	3.8 (0.53)	3.73 (0.51)	3.86 (0.57)	0.598

BMI: body mass index; LVEF: left ventricular ejection fraction; OPCAB: off-pump coronary artery bypass graft; ONCAB: on-pump coronary artery bypass graft; DM: diabetes mellitus; HT: hypertension; IQR: interquartile range; SD: standard deviation

Intraoperative and postoperative data are summarized in **Table 2**. No significant differences were found between OPCAB and ONCAB outcomes. While the ONCAB group showed trends of higher postoperative complication rates, longer ventilator support, ICU stays, and hospital stays, none were statistically significant. Additionally, there were no low cardiac output, perioperative myocardial infarctions, or in-hospital mortalities in either group.

**Table 2:** Intraoperative and postoperative data

Outcome	Total (n = 20)	OPCAB (n = 10)	ONCAB (n = 10)	P-value
Number of anastomoses; median (IQR)	3 (3, 3.5)	3.5 (3, 5)	3 (3, 3)	0.172
Number of arterial grafts (IQR)	1 (1, 2)	1 (1, 2)	1 (1, 2)	0.151
Number of vein grafts (IQR)	2 (1, 2)	2 (1, 2)	2 (1, 2)	0.264
AOX time (min); mean (SD)	62.6 (12.34)	–	62.6 (12.34)	–
CPB time (min); mean (SD)	115.1 (16.78)	–	115.1 (16.78)	–
Operative time (min); mean (SD)	274.25 (61.50)	285.10 (85.47)	263.40 (28.96)	0.457
Intra operative defibrillation (%)	1 (5.00)	–	1 (10.00)	>0.999
IABP insertion (%)	1 (5.00)	1 (10.00)	–	>0.999
Fever (%)	2 (10.00)	–	2 (20.00)	0.474
Stroke (%)	1 (5.00)	–	1 (10.00)	>0.999
AKI (%)	5 (25.00)	3 (30.00)	2 (20.00)	>0.999
Infection (%)	5 (25.00)	1 (10.00)	4 (40.00)	0.303
Ventilator >48 hours (%)	3 (15.00)	–	3 (30.00)	0.087
ICU stay (days); median (IQR)	4 (2, 6)	3 (2, 5)	4.5 (4, 7)	0.125
Hospital stay (days); mean (SD)	16.2 (6.93)	15.3 (5.52)	17.1 (8.32)	0.576

OPCAB: off-pump coronary artery bypass graft; ONCAB: on-pump coronary artery bypass graft; AOX: aortic cross-clamp; CPB: cardiopulmonary bypass; IABP: intra-aortic balloon pump; AKI: acute kidney injury; IQR: interquartile range; SD: standard deviation; ICU: intensive care unit

**Fig. 2:**
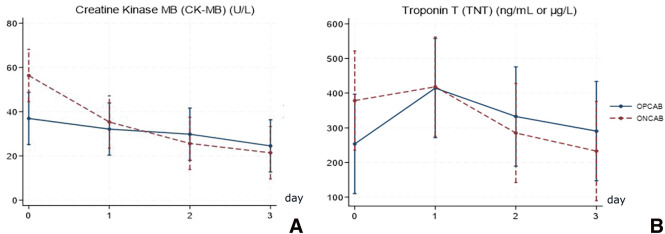
Linear graph predictions of CK-MB (**A**) and troponin T (**B**) levels from the GEE model, comparing over 4-time points: postoperative Days 0–3. CK-MB: creatine kinase MB; GEE: generalized estimating equations

There were no significant differences observed in terms of inotropic drug support and creatinine levels postoperatively using GEE analysis (**Table S1**). However, the graphical trend (**Fig. S1**) showed the ONCAB group had higher initial requirements for inotropic drugs such as dobutamine and adrenaline, while the OPCAB group required higher doses of norepinephrine in the early postoperative period. ONCAB patients exhibited significantly higher levels of CK-MB on postoperative Day 0 compared to OPCAB patients (P = 0.006). Troponin T levels also peaked higher in the ONCAB group, although this difference was not significant (**Fig. 2**).

### Metabolomics study results

**Fig. 3:**
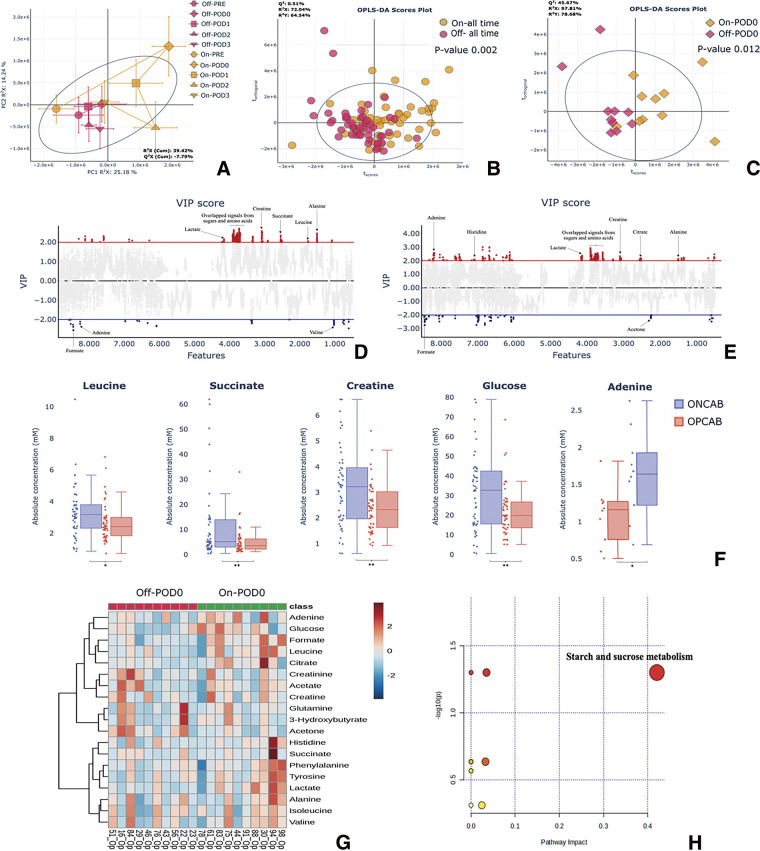
Key analyses of metabolomic data. (**A**) Time trajectory plot of ^1^H-NMR spectral data comparing ONCAB (yellow) and OPCAB (pink) groups across all time points. Data are presented as average principal component score ± SD. (**B**) OPLS-DA score plots comparing ONCAB vs. OPCAB at all time points, showing distinct clustering with a significant P-value of 0.002. (**C**) OPLS-DA score plots for ONCAB vs. OPCAB on postoperative Day 0, indicating a significant difference with a P-value of 0.012. (**D** and **E**) VIP score plots showing differential metabolites between ONCAB vs. OPCAB at all time points and postoperative Day 0, respectively. (**F**) Boxplots of significant differential metabolite concentrations comparing ONCAB vs. OPCAB at all time points and postoperative Day 0, indicating statistical significance (*P <0.05, **P <0.02). (**G**) Heatmap illustrating the relative abundance of differential metabolites, highlighting upregulated and downregulated trends between ONCAB vs. OPCAB on postoperative Day 0. (**H**) Summary of pathway analysis on differential metabolites between ONCAB vs. OPCAB on postoperative Day 0. ^1^H-NMR: proton nuclear magnetic resonance; OPLS-DA: orthogonal partial least squares discriminant analysis; VIP: variable importance in projection; CABG: coronary artery bypass graft; ONCAB: on-pump CABG; OPCAB: off-pump CABG; SD: standard deviation

Principal component analysis (PCA) was performed for the identification and interpretation of metabolite signals in the ^1^H-NMR spectra. Quality control samples within the 95% confidence interval confirmed data reliability. The PCA score plot stratified samples into ONCAB and OPCAB groups and the time trajectory plots (**Fig. 3A**) show metabolic shifts over time for both groups, with the ONCAB group exhibiting more pronounced metabolomic changes postsurgery compared to the OPCAB group.

### Intragroup analysis

We used orthogonal partial least squares discriminant analysis (OPLS-DA) to compare the metabolomic profiles between preoperative and postoperative Days 0–3 in the ONCAB group (**Fig. S2**). Although the analysis suggested potential differences, particularly on Day 0, the permutation tests confirmed no significant differences at any time point.

Similarly, for the OPCAB group, OPLS-DA and permutation tests (**Fig. S3**) revealed no significant differences in metabolomic profiles between the preoperative period and any postoperative time points.

### Intergroup analysis

We conducted OPLS-DA comparisons between the ONCAB and OPCAB groups across all time points. We observed clear clustering and good discrimination (**Fig. 3B**), as confirmed by permutation tests showing a significant P-value of 0.002. Further analysis of metabolomic profiles between the groups at each specific time point (**Fig. S4**) revealed distinct separation on postoperative Day 0 (**Fig. 3C**), confirmed by permutation tests with a significant P-value of 0.012. Comparisons at other time points showed no significant differences.

### Differential metabolites identification

The variable importance in projection (VIP) score analysis was used to identify differential metabolites contributing to differences in metabolomic profiles. Metabolites with VIP scores above 2 were considered significant. The VIP score plots comparing ONCAB and OPCAB at all time points (**Fig. 3D**) and specifically on postoperative Day 0 (**Fig. 3E**) highlighted significant differential metabolites, including creatine, succinate, leucine, valine, alanine, adenine, histidine, citrate, and acetone.

When we proceeded with univariate analysis, it revealed significant differences in concentrations only in leucine, succinate, creatine, glucose, and adenine between the groups, all of which were higher in the ONCAB group (**Figs. 3F** and **3G**). Pathway analysis also indicated significant perturbations in starch and sucrose metabolism (**Fig. 3H**).

### The correlation between targeted metabolites and cardiac enzyme profiles

**Fig. 4:**
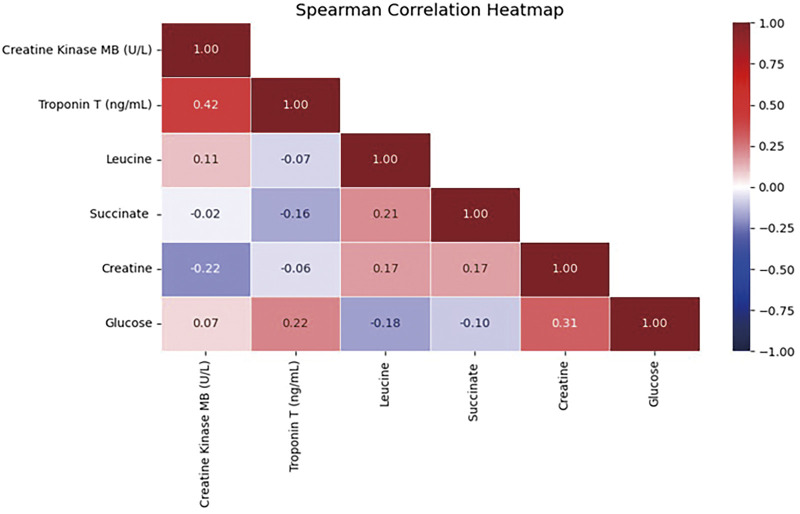
The Spearman correlation coefficients (ρ) between cardiac enzymes (Troponin T and CK-MB) and differential metabolites. CK-MB: creatine kinase MB

To investigate the correlation between cardiac enzymes and significant differential metabolites, we employed the Spearman correlation test, with the results depicted in **Fig. 4**. This analysis revealed that there were no significant relationships between cardiac enzymes and the targeted metabolites.

## Discussion

Our results indicate that both ONCAB and OPCAB procedures are associated with postoperative metabolomic shifts, with ONCAB exhibiting a more pronounced effect. We confirmed significant differences in metabolomic profiles between the techniques, particularly in the early postoperative period.

### Clinical outcomes and cardiac enzyme level

In our study, we found no significant difference in postoperative clinical outcomes between the groups. However, the OPCAB group exhibited a lower rate of postoperative complications and shorter ICU and hospital stays, aligning with other studies.^7)^

Several studies have highlighted the potential advantages of OPCAB in reducing cardiac enzyme levels postsurgery.^8)^ In our investigation, we observed significantly lower levels of peak CK-MB on postoperative Day 0 in the OPCAB, while troponin T levels showed no significant differences. This finding is consistent with previous research.^9^^,^^10)^

### Metabolomic profile according to type of surgery

Our study identified distinct patterns in metabolomic profiles between preoperative and postoperative periods in both techniques, particularly in the immediate postsurgery, although not statistically significant. The ONCAB group showed a greater degree of metabolomic disruption compared to OPCAB.

In intergroup analysis, significant differences in metabolomic patterns between ONCAB and OPCAB groups were observed on postoperative Day 0, which normalized in the subsequent days. This pattern, consistent with previous findings,^11)^ underscores the importance of early postoperative care in optimizing patient outcomes.

### Metabolomic perturbation and clinical implications

**Fig. 5:**
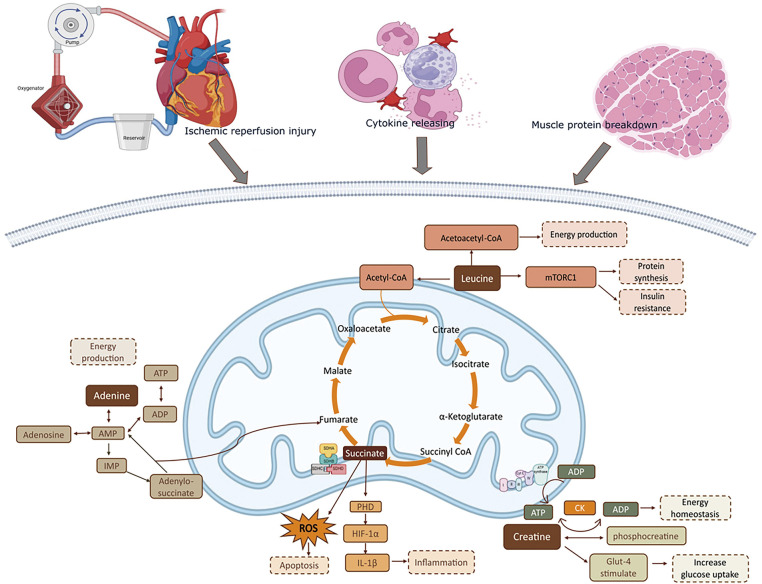
Highlights the clinical implications of the study findings. **Leucine** fuels energy production via acetoacetate and acetyl-CoA in the TCA cycle, activates mTORC1 for protein synthesis, and influences insulin resistance. **Creatine** forms creatine phosphate for rapid cell energy. **Succinate** stabilizes HIF-1α, triggering IL-1β and ROS in hypoxia. **Adenine** drives ATP regulation. mTORC1: mammalian target of rapamycin Complex 1; ATP: adenosine triphosphate; ADP: adenosine diphosphate; CK: creatine kinase; Glut-4: glucose transporter type 4; ROS: reactive oxygen species; PHD: prolyl hydroxylase domain (proteins); HIF-1α: hypoxia-inducible factor 1-alpha; IL-1β: interleukin-1 beta; AMP: adenosine monophosphate; IMP: inosine monophosphate. Created with BioRender.com

We identified differential metabolites contributing to the profile differences between groups. These include leucine, succinate, creatine, glucose, and adenine (**Fig. 5**).

Leucine, a branched-chain amino acid (BCAA) essential for protein synthesis and energy production, may indicate muscle breakdown under stress. In our study, elevated leucine levels in the ONCAB group suggest increased muscle catabolism, likely triggered by the inflammatory response and ischemia-reperfusion injury associated with CPB. Although more arterial grafts could increase ischemia, we found no significant difference in graft usage between ONCAB and OPCAB, indicating that the elevated protein degradation markers are likely due to the metabolic burden of CPB. Moreover, leucine can slow muscle degradation by boosting protein synthesis,^12)^ but high levels are also associated with insulin resistance through mTORC1 activation and mitochondrial dysfunction.^13^^,^^14)^ Additionally, leucine catabolism into ketone bodies inhibits cardiac glucose uptake by reducing GLUT4 translocation, altering energy metabolism, and contributing to oxidative stress and impaired cardiac function.^15)^ Some studies have found that dietary supplements in animal and human models can improve BCAA metabolism and insulin sensitivity, suggesting a strategy to treat cardiac failure. ^12^^,^^16)^

Succinate, an intermediate in the tricarboxylic acid (TCA) cycle critical for cellular energy production and influencing inflammation and oxidative stress,^17)^ shows higher levels in the ONCAB group, likely in response to the effects of CPB. During ischemia, succinate accumulates and, upon reperfusion, rapidly oxidizes via succinate dehydrogenase (SDH), triggering reactive oxygen species (ROS) production by the mitochondrial complex.^18)^ This ROS generation contributes to ischemia-reperfusion injury and metabolic stress. Inhibiting succinate accumulation has shown promise in murine heart attack models,^18^^,^^19)^ with malonate effectively inhibiting SDH and reducing reperfusion injury.^20)^

Creatine is essential for rapid adenosine triphosphate (ATP) generation and acts as a critical high-energy phosphate buffer in tissues with high metabolic demands like myocytes. Changes in creatine levels reflect altered energy demand during surgical stress. It stabilizes mitochondria, reduces reactive oxygen species production, and supports glucose metabolism for energy storage and uptake. Studies highlight its benefits in heart failure, with exogenous creatine phosphate showing cardioprotective effects in ischemic heart disease.^21^^,^^22)^ Additionally, creatine maintains cellular osmolarity and regulates pH levels, and its dysregulation can worsen tissue injury and inflammation.^23)^

Glucose, the primary cellular energy source, was significantly elevated in ONCAB patients in our study. Pathway analysis highlighted disruptions in sugar and starch metabolism, indicating altered glucose handling and potential stress hyperglycemia linked to CPB.^24)^ This rise may result from surgical stress-induced insulin resistance and inflammation,^25)^ reflecting an adaptive response to maintain sufficient energy supply under stress. Managing glucose levels is crucial, as stress-induced hyperglycemia can adversely affect recovery and outcomes in cardiac surgery patients.^26)^

Adenine is essential for forming ATP and serves as a precursor for adenosine and other molecules.^27)^ Elevated adenine levels in ONCAB patients suggest altered nucleotide metabolism and cellular stress, indicating higher ATP turnover or breakdown due to increased energy demands and tissue damage during surgery. This rise could also result from nucleotide release during reperfusion.^28)^ Exogenous adenosine supplementation can restore the adenine nucleotide pool after ischemic injury. Additionally, adenine sulfate has been reported to preserve cardiac function and the cholinergic system against myocardial infarction by increasing nitric oxide and nitric oxide synthase activity, offering cardioprotective effects against ischemia-reperfusion injury.^29)^

### Correlation between metabolic perturbation and clinical outcomes

Our study found no clinical differences between the two techniques despite significant differences in their metabolomic profiles. This result may be due to the small sample size and the inclusion of low-risk patients. However, studying high-risk or borderline patients could reveal different outcomes. Turer et al.^30)^ found that postsurgery metabolic changes are closely correlated with hemodynamic fluctuations, particularly in high-risk patients, such as those with left ventricular dysfunction, suggesting the potential value of perioperative metabolic monitoring and tailored optimization strategies to improve cardiac surgical outcomes.

It is important to note that our findings indicated greater metabolomic perturbations in the ONCAB group, highlighting potential strategies to mitigate these shifts. These strategies include optimizing CPB management and cardioplegia solutions, which are essential for ensuring adequate tissue perfusion and oxygen delivery. Innovative approaches, such as biocompatible circuit coatings, may help reduce inflammation and metabolic stress. Close postoperative monitoring is crucial for detecting early metabolic disturbances, particularly the glucose fluctuations observed during ONCAB, underscoring the need for targeted glucose management and enhanced nutritional support, which could involve specific supplementation. Additionally, pharmacological interventions, including antioxidants and metabolic modulators, may aid in stabilizing metabolism during and after surgery.

Furthermore, our results emphasize the metabolic advantages associated with OPCAB, suggesting it may be preferable for patients with higher metabolic risks, including older adults and individuals with metabolic diseases or heart failure, who could benefit from the OPCAB approach.

## Limitation

Our study has several limitations. First, the sample size, though calculated based on prior studies to meet the minimum threshold for sufficient power, remains small due to its pilot nature and resource constraints. This may have hindered our ability to detect significant differences, particularly for less abundant metabolites or those with smaller effect sizes. Furthermore, inherent variations among patients, which are common in complex metabolomic studies, could serve as confounding factors that influence the outcomes. Future studies with larger sample sizes, refined methodologies, and a focused analysis of key metabolites will help clarify the clinical significance of these metabolic shifts and guide targeted interventions to optimize patient outcomes.

## Conclusion

Our study found no significant differences in clinical outcomes between the groups; however, the ONCAB group exhibited higher peaks of cardiac enzymes. Both techniques resulted in postoperative metabolomic perturbations, with ONCAB having a greater impact. Significant differences in metabolomic profiles were observed, particularly on postoperative Day 0. Key metabolites included leucine, succinate, creatine, adenine, and glucose, with pathway analysis indicating notable effects on starch and sucrose metabolism. These changes suggest increased protein and energy turnover, mitochondrial stress, altered glucose metabolism, and heightened cellular stress responses. Understanding these alterations is crucial for developing targeted interventions to improve patient outcomes, particularly for patients at higher metabolic risk who may benefit from off-pump CABG.

## Acknowledgments

We appreciate CARI KKU and the Esarn Heart and Lungs Research group for their assistance in specimen collection.

## Declarations

### Ethical statements

The study protocol was thoroughly reviewed and approved by the Institutional Review Board (IRB) and the Ethical Committee of the Center for Ethics in Human Research at the Faculty of Medicine, Khon Kaen University (IRB number: HE651465). Written informed consent was obtained from all participating patients to ensure compliance with ethical standards and regulatory requirements.

### Funding

This study was granted by the Faculty of Medicine, Khon Kaen University, Thailand (grant number: IN66030).

### Data availability statement

The data underlying this article will be shared on reasonable request to the corresponding author.

### Author contributions

Chananya Karunasumetta: Conducted experiments and wrote the main manuscript.

Wijittra Tourthong and Rachata Mala: Involved in experiments and patient recruitment.

Chotika Chatgasem and Theerayut Bubpamala: Contributed to data acquisition, statistical analyses, and graphics.

Suriya Punchai and Kittisak Sawanyawisuth: Conceived the work and provided manuscript consultation.

All authors have reviewed and approved the final version of the manuscript.

### Conflicts of interest

The authors declare no conflicts of interest associated with this manuscript.

## Supplementary Material

Table S1 Generalized Estimating equations-based regression analysis of postoperative outcomes comparing ONCAB and OPCAB

Fig. S1 Linear graph predictions from GEE analysis, comparing postoperative outcomes between ONCAB and OPCAB techniques for various parameters over 4 days (postoperative Days 0 to 3). The variables include (**A**) dobutamine dosage requirement, (**B**) adrenaline requirement, (**C)** norepinephrine requirement, (**D**) postoperative creatinine levels, (**E**) CK-MB levels, and (**F**) TNT levels. The y-axis represents the levels of inotropic drugs, creatinine, and cardiac enzymes, while the x-axis denotes time across postoperative Days 0, 1, 2, and 3. This model effectively accounts for multiple observations, providing a detailed temporal comparison of the postoperative effects of ONCAB and OPCAB. GEE: generalized estimating equations; CK-MB: creatine kinase-MB; TNT: troponin T; CABG: coronary artery bypass graft ONCAB: on-pump CABG; OPCAB: off-pump CAB

Fig. S2 The OPLS-DA score plots derived from ^1^H-NMR spectral data highlight the metabolomic profiles of patients undergoing ONCAB surgery. These plots compare the preoperative period with postoperative Days 0 (**A**), 1 (**B**), 2 (**C**), and 3 (**D**). OPLS-DA: orthogonal projections to latent structures discriminant analysis; ^1^H-NMR: proton nuclear magnetic resonance spectroscopy; ONCAB: on-pump coronary artery bypass graft

Fig. S3 Demonstrating OPLS-DA score plots of patients undergoing OPCAB. The comparison was conducted between the preoperative period and postoperative Day 0 (**A**), Day 1 (**B**), Day 2 (**C**), and Day 3 (**D**). OPLS-DA: orthogonal projections to latent structures discriminant analysis; OPCAB: off-pump coronary artery bypass graft

Fig. S4 Demonstrating OPLS-DA score plots derived from ^1^H-NMR spectral data, comparing patients undergoing on-pump and off-pump CABG. The comparison was conducted between the preoperative period (**A**) and postoperative Day 0 (**B**), Day 1 (**C**), Day 2 (**D**), and Day 3 (**E**). OPLS-DA: orthogonal projections to latent structures discriminant analysis; ^1^H-NMR: proton nuclear magnetic resonance spectroscopy; CABG: coronary artery bypass graft
