# Supplementary material for: Comparative Analysis of Metabolomic Responses in On-Pump and Off-Pump Coronary Artery Bypass Grafting
Source: Ann Thorac Cardiovasc Surg. 2024 Dec 5;30(1):24-00126. doi: 10.5761/atcs.oa.24-00126 (PMC11634389; doi:10.5761/atcs.oa.24-00126)
Supplement: Table S1 [file atcs-30-1-24-00126-s01.pdf]

Supplement Table 1: Generalized Estimating Equations-Based Regression Analysis of Postoperative Outcomes Comparing ONCAB and OPCAB

	**Coefficient**	**95%CI**	**p-value**
**Dobutamine (ug/kg/min)**			
Post operative day 0	0.67	-0.35, 1.69	0.409
day 1	0.2	-0.82, 1.22	>0.999
day 2	-0.3	-1.32, 0.72	>0.999
day 3	-0.3	-1.32, 0.72	>0.999
**Adrenaline (ug/kg/min)**			
Post operative day 0	0.005	0, 0.01	0.275
day 1	-0.002	-0.01, 0	>0.999
day 2	0.001	-0.01, 0.01	>0.999
day 3	1.73E-18	-0.01, 0.01	>0.999
**Levophed (ug/kg/min)**			
Post operative day 0	-0.02	-0.05, 0.01	0.647
day 1	-0.002	-0.04, 0.03	>0.999
day 2	0.01	-0.02, 0.04	>0.999
day 3	0.006	-0.03, 0.04	>0.999
**Creatinine (mg/dl)**			
Post operative day 0	-0.11	-1.1, 0.88	>0.999
day 1	0.17	-0.82, 1.17	>0.999
day 2	0.33	-0.67, 1.32	>0.999
day 3	0.39	-0.61, 1.38	>0.999
**CK-MB (ng/ml)**			
Post operative day 0	19.40	4.15, 34.65	0.006
day 1	3.20	-12.05, 18.45	>0.999
day 2	-4.20	-19.45, 11.05	>0.999
day 3	-3.10	-18.35, 12.15	>0.999
**Troponin T (ng/dl)**			
Post operative day 0	125.07	-59.85, 309.99	0.365
day 1	3.80	-181.12, 188.72	>0.999
day 2	-47.60	-232.52, 137.32	>0.999
day 3	-57.70	-242.62, 127.22	>0.999
