# Supplementary material for: Comparative Analysis of Metabolomic Responses in On-Pump and Off-Pump Coronary Artery Bypass Grafting
Source: Ann Thorac Cardiovasc Surg. 2024 Dec 5;30(1):24-00126. doi: 10.5761/atcs.oa.24-00126 (PMC11634389; doi:10.5761/atcs.oa.24-00126)
Supplement: Fig. S1 [file atcs-30-1-24-00126-s02.pdf]

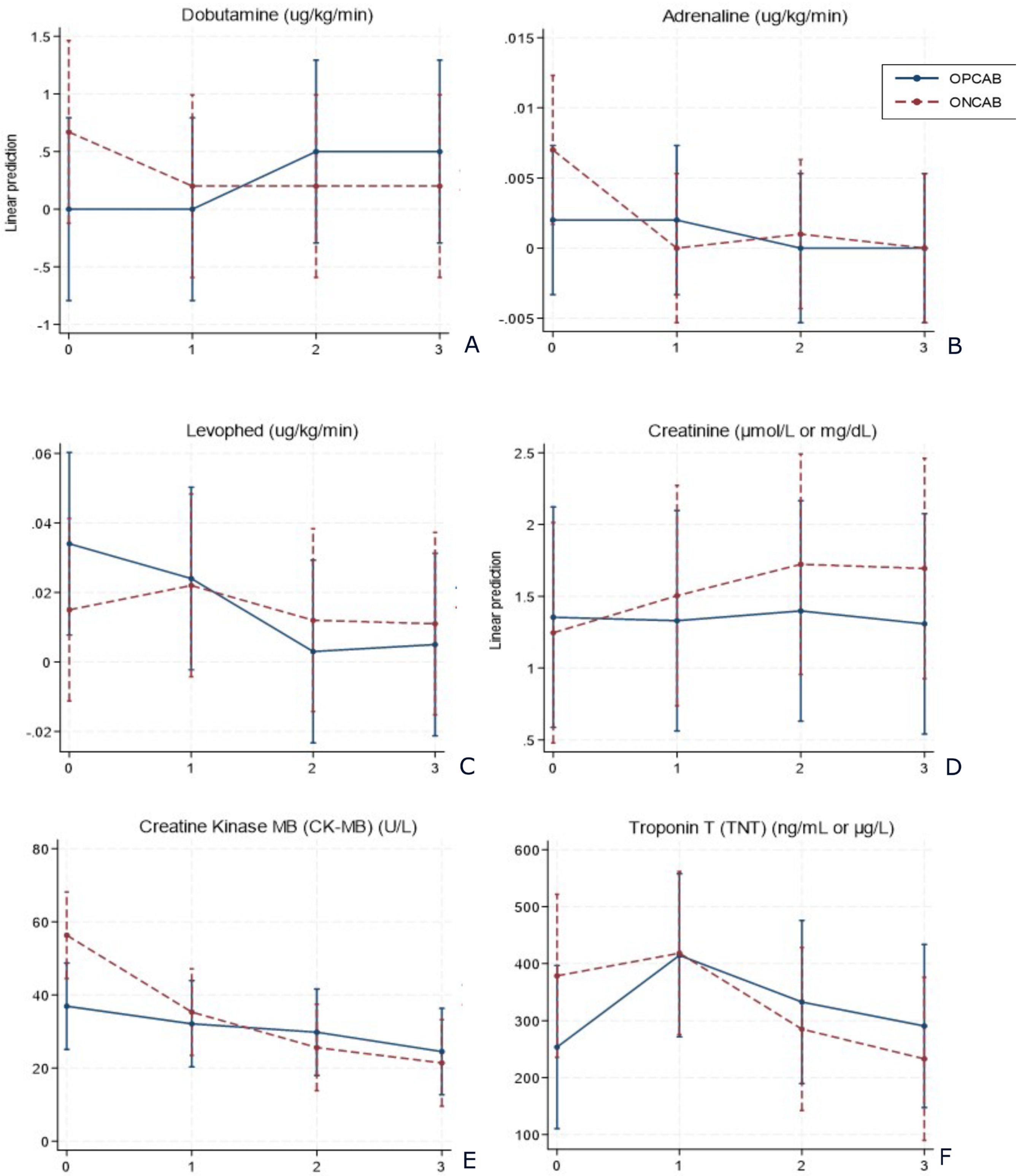

Supplement Figure 1: Linear Graph Predictions from Generalized Estimating Equations (GEE) Analysis Comparing Postoperative Outcomes between ONCAB and OPCAB Techniques for Various Parameters Over Four Days (Postoperative Days 0 to 3). The variables include (A) dobutamine dosage requirement, (B) adrenaline requirement, (C) Levophed requirement, (D) postoperative creatinine levels, (E) creatine kinase-MB (CK-MB) levels, and (F) troponin T (TNT) levels. The y-axis represents the levels of inotropic drugs, creatinine, and cardiac enzymes, while the x-axis denotes time across postoperative days 0, 1, 2, and 3. This model effectively accounts for multiple observations, providing a detailed temporal comparison of the postoperative effects of ONCAB and OPCAB.
