# Supplementary material for: Comparative Analysis of Metabolomic Responses in On-Pump and Off-Pump Coronary Artery Bypass Grafting
Source: Ann Thorac Cardiovasc Surg. 2024 Dec 5;30(1):24-00126. doi: 10.5761/atcs.oa.24-00126 (PMC11634389; doi:10.5761/atcs.oa.24-00126)
Supplement: Fig. S2 [file atcs-30-1-24-00126-s03.pdf]

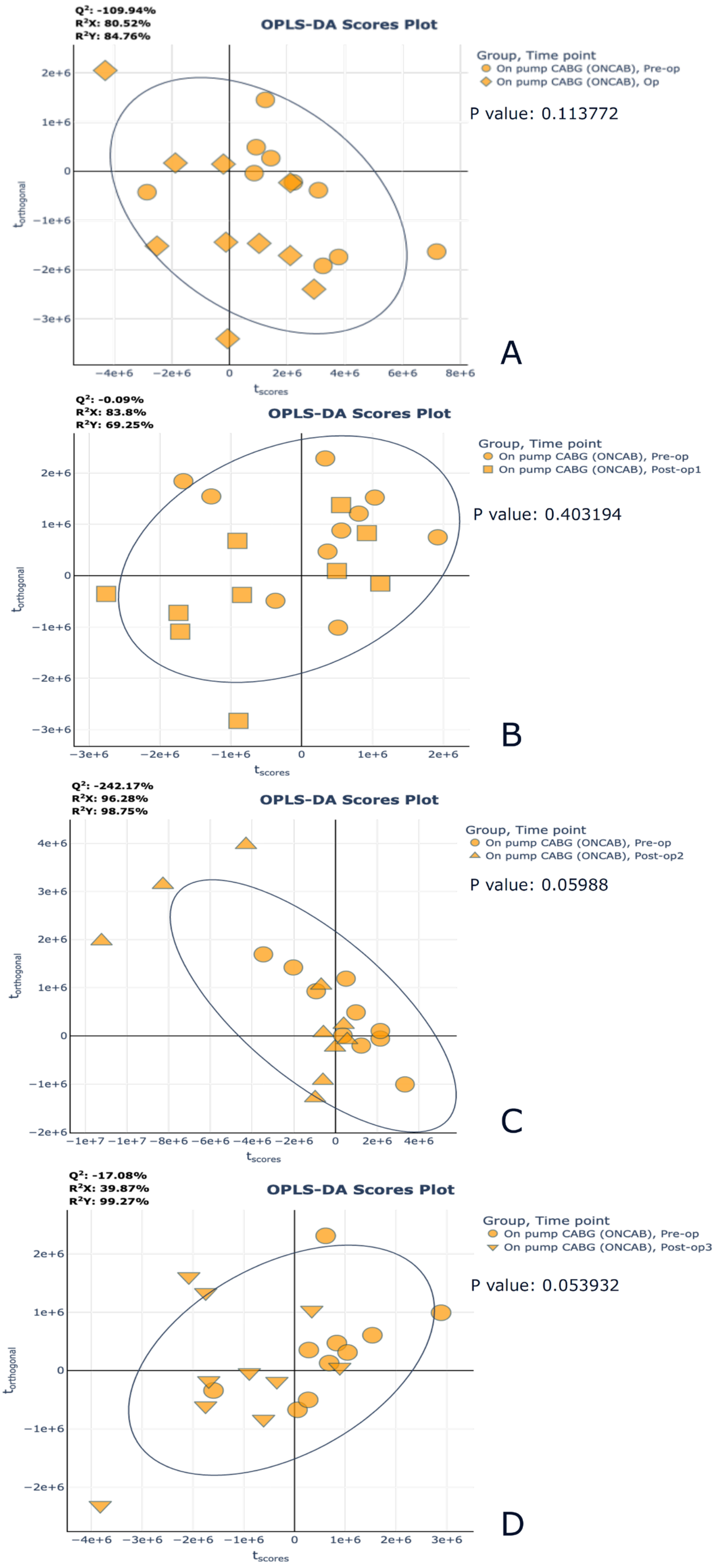

Supplement Figure 2 The OPLS-DA score plots derived from 1H NMR spectral data highlight the metabolomic profiles of patients undergoing on-pump coronary artery bypass graft (ONCAB) surgery. These plots compare the pre-operative period with post-operative days 0 (A), 1 (B), 2 (C), and 3 (D). (OPLS-DA: Orthogonal Projections to Latent Structures Discriminant Analysis; 1H NMR: proton nuclear magnetic resonance spectroscopy)
