# Supplementary material for: Comparative Analysis of Metabolomic Responses in On-Pump and Off-Pump Coronary Artery Bypass Grafting
Source: Ann Thorac Cardiovasc Surg. 2024 Dec 5;30(1):24-00126. doi: 10.5761/atcs.oa.24-00126 (PMC11634389; doi:10.5761/atcs.oa.24-00126)
Supplement: Fig. S4 [file atcs-30-1-24-00126-s05.pdf]

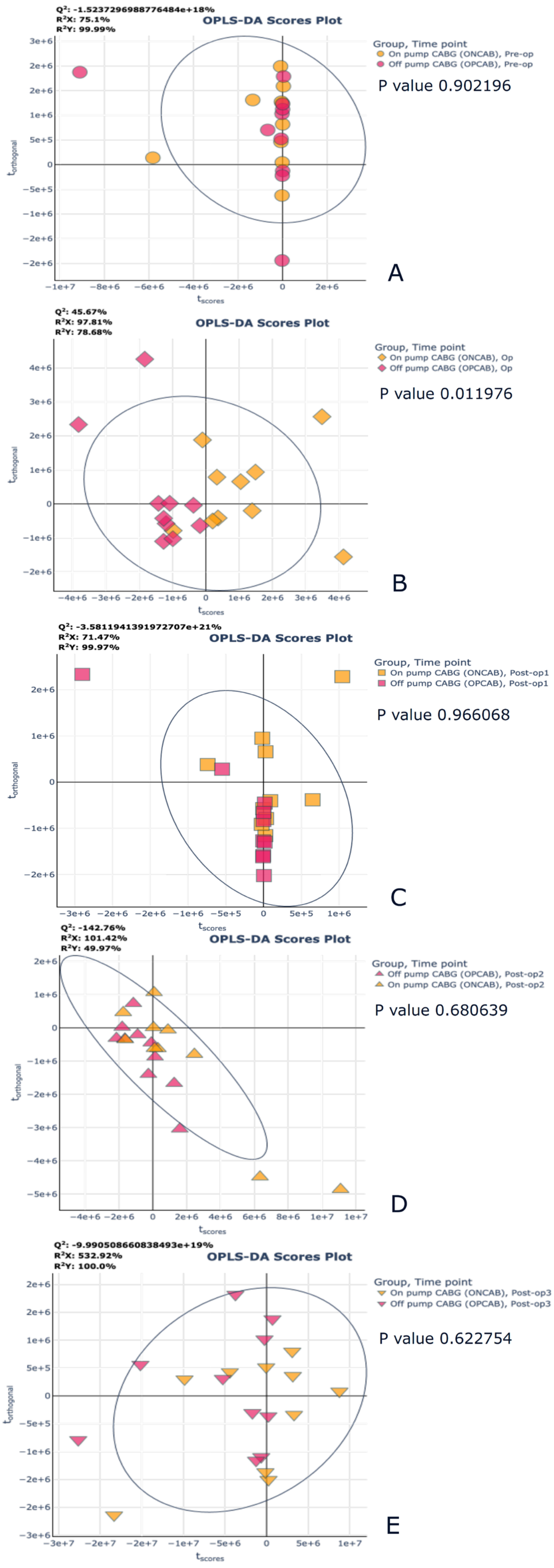

Supplement Figure 4 demonstrates OPLS-DA score plots derived from 1H NMR spectral data of comparing of patients undergoing on-pump and off-pump CABG. The comparison was conducted between the pre-operative period (A) and post-operative day 0 (B), day 1 (C), day 2 (D) and day 3 (E)
